# The Risks Associated with Inhalation Exposure to Cosmetics and Potential for Assessment Using Lung Organoids

**DOI:** 10.3390/bioengineering12060652

**Published:** 2025-06-13

**Authors:** Yiguang Li, Xin Luo, Rong Hu, Lifeng Tang, Qi Xiang

**Affiliations:** 1 State Key Laboratory of Bioactive Molecules and Druggability Assessment, Jinan University, Guangzhou 510632, China; yiguangli@126.com; 2 Biopharmaceutical R&D Center of Jinan University Co., Ltd., Guangzhou 510632, China; luoxin@stu2023.jnu.edu.cn; 3 Guangdong Provincial Key Laboratory of Bioengineering Medicine, Institute of Biomedicine, Jinan University, Guangzhou 510632, China; 4 HBN Research Institute and Biological Laboratory, Shenzhen Hujia Technology Co., Ltd., Shenzhen 510663, China; rong_114819@163.com; 5 Guangzhou Xika Technology Co., Ltd., Guangzhou 510330, China; tanglifeng@tuiquan.com

**Keywords:** cosmetics, inhalation exposure, lung organoids, risk assessment

## Abstract

This review addresses the exposure risks associated with the inhalation of aerosolized cosmetic products and explores the utility of lung organoids in assessing these risks. Aerosolized cosmetics such as sprays pose potential health hazards through inhalation, necessitating a thorough evaluation of exposure levels. Traditional methods for assessing inhalation risks have limitations, prompting the exploration of more sophisticated models. Lung organoids, three-dimensional structures derived from stem cells, offer a biologically relevant model for studying lung responses to inhaled substances. This review discusses the construction of lung organoids, their characteristics, and the advantages that they provide over conventional models. Furthermore, it examines existing studies that have employed lung organoids to evaluate the effects of cosmetic inhalation exposure, highlighting the potential of this approach to enhance the safety assessments of cosmetic products. We aim to establish lung organoids as a reliable tool for future research, ensuring the safety and regulatory compliance of cosmetics.

## 1. Introduction

Cosmetics are defined as chemical industrial products for daily use that are applied to the skin, hair, nails, lips and other surfaces of the human body for cleansing, protecting, beautifying, and modifying through methods such as rubbing and spraying. Cosmetics are available in various dosage forms, including creams, ointments, milks, and liquids. Additionally, sprays, powders, and other forms of cosmetics are prevalent. Before being marketed, cosmetics must undergo a series of safety tests to ensure their safety for human use. These tests include hazard identification, dose–response assessment, and exposure assessment. Historically, animal testing was a commonly employed assessment method; however, to protect animal rights, several countries and regions have advocated for prohibiting or reducing animal testing. Consequently, there is a pressing need to identify effective alternatives.

With the advancement of technologies such as cell biology and tissue engineering, organoids have emerged as a promising alternative model for various biological studies and safety assessments. Research has been undertaken to assess the efficacy of these products by applying them to in vitro reconstituted skin models and measuring their oxygen radical uptake capacity [1]. Skin organoids offer a more accurate representation of the physiological state of an organism compared to traditional experiments utilizing two-dimensional (2D) skin cells. Currently, the safety evaluation of cosmetics utilizing organoid models predominantly focuses on dermal exposure, specifically assessing the effects on skin tissue. However, spray cosmetics present extra inhalation exposure risks that can adversely affect the respiratory system. Research addressing the consequences of inhalation exposure remains largely underexplored. Furthermore, to date, there has been no comprehensive exploration into the toxicity and potential hazards associated with inhalation exposure as evaluated through the application of respiratory system organoids. This gap highlights a critical need for systematic investigations that leverage respiratory organoid models to elucidate the effects of aerosolized cosmetic products on lung health. By addressing this overlooked area of study, future research could provide valuable insights that enhance the safety evaluation frameworks for cosmetics, thereby contributing to improved regulatory standards and consumer safety.

This study will provide a comprehensive evaluation of the safety issues associated with inhalation exposure, specifically focusing on the risks related to cosmetic safety. Additionally, the feasibility of utilizing lung organoids for the safety assessment of cosmetic products intended for respiratory exposure will be examined.

## 2. Inhalation Exposure

Assessing cosmetics’ safety risks encompasses evaluating the inherent toxicity of the raw materials as well as the potential effects on the human body resulting from exposure to these products. Certain raw materials utilized in cosmetics may possess toxic properties, or harmful by-products, such as dioxane, may be produced during the manufacturing process. The toxicity risks of cosmetics and their raw materials are mentioned in the Guidelines for the Safety Assessment of Cosmetics. These risks, as detailed in the aforementioned guidelines, include various categories, such as acute toxicity, irritation/corrosivity, phototoxicity, genotoxicity, repeated-dose toxicity, reproductive and developmental toxicity, and chronic toxicity. These toxicity risks are typically assessed in conjunction with dosage considerations. Although advancements in regulatory frameworks have led to a decrease in incidents of cosmetic toxicity, the potential risks associated with long-term use remain challenging to ascertain within the safety assessment process and require further investigation.

Cosmetics are primarily applied to human skin, and adverse reactions resulting from their use are a significant concern. Among these reactions, cosmetic contact dermatitis is the most prevalent, and it can be categorized into two types: irritant contact dermatitis (ICD) and allergic contact dermatitis (ACD) [2]. Cosmetic contact dermatitis is typically triggered by the ingredients present in cosmetic products, such as fragrances and preservatives. Additionally, the application of cosmetics on already compromised skin or improper usage of these products can precipitate this condition [3]. Cosmetic contact dermatitis generally manifests at the site of application, with the facial area being the most frequently affected. Common clinical presentations include erythema, papules, and edema [4].

Inhalation exposure arises when aerosolized or powdered cosmetics are inhaled through the oral cavity or respiratory tract during application. Loose powder cosmetics present a greater risk of inhalation exposure compared to compact powder formulations [5]. However, in practical terms, spray cosmetics are more commonly used and, consequently, exhibit a higher probability of causing inhalation exposure.

Spray cosmetics can be categorized into aerosols and sprays. The distinction between these two lies in their respective product mechanisms, resulting in the formation of droplets with diameters predominantly less than 10 μm. In contrast, sprays employ a mechanical pump to generate pressure, which leads to the production of larger droplets, with an average diameter of approximately 70 μm [6]. The diameter of particulate matter (PM) is significant, as it influences the deposition sites within the respiratory tract; smaller particles tend to deposit closer to the lung (Table 1) [7,8]. There is a notable disparity in the particle diameters produced by aerosols and sprays, with aerosols yielding smaller particles. This difference in particle size correlates with varying deposition sites within the respiratory system for the two types of spray mechanisms. Significant differences exist in the inhalable particle characteristics among various formulations of propellant and pump sprays. The proportion of inhalable droplets and particles released by propellant sprays varies substantially across formulations. Specifically, the percentages of inhalable particles are as follows: emulsions at 0.15%, lacquers at 7.5%, oils at 0.68%, and powders at 23.5% [8]. In contrast, pump sprays exhibit a markedly lower proportion of inhalable particles across all formulations, with values remaining below 1%. The percentages for pump sprays are emulsions at 0.0%, lotions at 0.8%, oils at 0.8%, and water-based formulations at 0.5% [8]. Regardless of the method employed for spray generation, it is essential to assess the particle size distribution and other aerosol characteristics, as well as their temporal changes, including clumping, sedimentation, and aging effects. Such comprehensive evaluations are crucial for conducting thorough safety assessments.

Water-soluble and hydrophilic compounds are typically retained within the mucous membranes of the upper respiratory tract, while more lipophilic and less water-soluble substances may penetrate deeper into the pulmonary system. Lung irritation caused by respirable particles can be categorized into two distinct types [9]: (i) sensory irritation, which involves the stimulation of nerve endings in the upper respiratory tract without resulting in detrimental alterations to lung tissue, and (ii) localized toxic effects, which lead to specific adverse changes in lung tissue. In instances where cosmetic raw materials or finished products contain toxic substances, these ingredients may be absorbed by the lungs and circulated throughout the body.

When assessing the safety of sprays in terms of inhalation exposure, it is essential to evaluate whether the various components of the sprays (Table 2) are allergenic. Sensitizing aerosol particulate matter that enters the respiratory tract can induce irritation, which may present as symptoms such as redness, swelling, itching or discomfort in the mucous membranes [10]. Following the inhalation of irritant chemicals, stimulated epithelial cells, leukocytes, and necrotic cells promptly release alarmins, while various multifunctional molecules promote the activation of innate immune cells and the recruitment and activation of antigen-presenting cells through pattern-recognition receptors. This cascade of events can result in inflammation, asthma, and other related disorders. Additionally, sensory nerves or non-neuronal cells may be activated through the stimulation of isolated chemosensory cells or via the direct activation of chemoreceptors, specifically transient receptor potential channels expressed in response to cellular injury, thereby promoting localized inflammation [11,12]. This phenomenon is commonly referred to as respiratory sensitization, and the agents responsible for this condition are known as respiratory sensitizers. In most instances, respiratory sensitization is triggered by high-molecular-weight antigens, with immunoglobulin E (IgE) and T helper cell (Th)2 subsets playing pivotal roles in this process [13,14], which is closely linked to the mechanisms of inflammation and sensitization. Conversely, low-molecular-weight chemicals, such as metal ions, typically bind to proteins to provoke an immune response in a process known as chemical respiratory sensitization.

In addition to the above-mentioned potential mechanisms underlying inhalation toxicity, the deposition of insoluble particles can contribute to lower respiratory toxicity. Localized lower respiratory toxicity is typically associated with the presence of insoluble particles. The lungs possess a specific defense mechanism designed to eliminate these insoluble particles, thereby preventing damage to both organs and organisms under conditions of low-to-moderate exposure. Macrophages play a crucial role in this process by internalizing and/or degrading the particles through phagocytosis, facilitating the removal of inhaled particles from the lungs and mitigating their interaction with lung tissue [9]. However, the capacity of macrophage-mediated clearance of insoluble inhaled particles is relatively limited, which may result in an overload when particulate concentrations are exceedingly high, potentially leading to chronic damage. It has been hypothesized [15] that this overload mechanism is attributable to the impaired mobility of alveolar macrophages following prolonged over-phagocytosis. Current research [16] indicates that this lung overload can precipitate chronic inflammation and fibrosis, and it may also contribute to the development of lung tumors in cases of long-term exposure to hazardous substances.

## 3. Safety Evaluation of Inhalation Exposure

### 3.1. Principles of Inhalation Exposure Assessments

**Figure 1:**
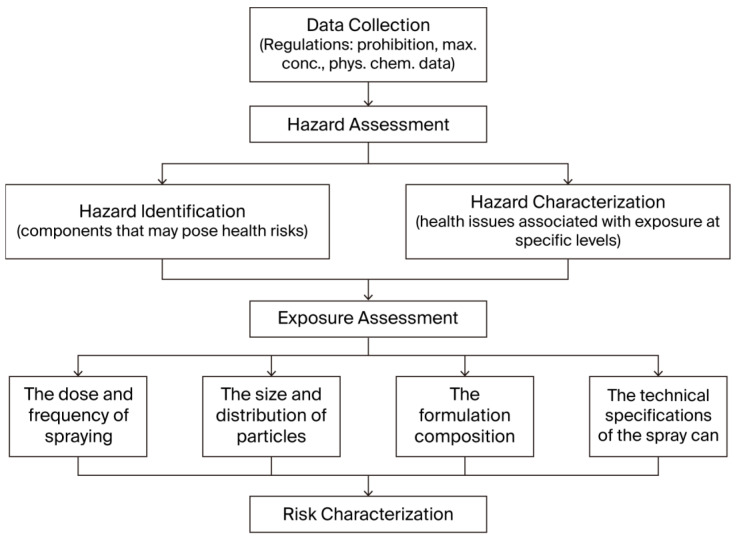
Route for safety assessment of inhalation exposure.

When there is potential for inhalation exposure, it is imperative to consider the health hazard effects associated with such exposure. Steiling et al. [5] proposed that the safety assessment of inhalation exposure encompass four key elements (Figure 1): (1) Data Collection: It is essential to evaluate the available safety data for all ingredients and their specific regulatory requirements. (2) Hazard Assessment: This process primarily involves hazard identification and hazard characterization. Hazard identification identifies components that may pose health risks, while hazard characterization evaluates the health issues associated with exposure at specific levels. (3) Exposure Assessment: Exposure estimates are determined by assessing the actual dose and frequency of spraying, along with the size and distribution of particles, the formulation composition, and the technical specifications of the spray can (e.g., nozzle design, dimensions, type of propellant). (4) Risk Characterization: Measured inhalation exposure data are compared to appropriate threshold values to assess risk.

The risk assessment of aerosolized products is inherently more complex due to the multitude of variables that influence exposure, as well as the characteristics of the particles and droplets released during a spraying event. The size and velocity of these particles and droplets are critical determinants of whether exposure is primarily localized in the upper respiratory tract or diffused in the alveolar region, which is contingent upon differential uptake by the inhalation route. Furthermore, the dimensions of the particles and droplets, along with the velocity of the spray, are affected by various technical parameters, including the pressure within the spray tank, the size of the tank and the geometry of the spray nozzle. Additionally, the components of the product, such as propellants and solvents, may trigger exposure events in specific regions of the respiratory tract. Given that the final exposure scenarios are influenced by these parameters and are often not directly comparable to the exposure scenarios employed in standard inhalation toxicity studies, there is a pressing need to develop more appropriate risk assessment methodologies to ensure the robust and reliable evaluation of risk.

### 3.2. Methods of Inhalation Exposure Evaluation

Real-time measurements of product-specific inhalation exposure represent the gold standard for exposure assessment; however, they necessitate complex study designs and are more frequently derived through mathematical methods. Data on exposure have been documented (Table 3). The concentration of any constituent in ambient air can be estimated based on the amount applied, the duration of application, and the volume of distribution, particularly in a situation where the sprayed particles are fully inhaled [17,18]. By employing conservative default values, risk thresholds may be overestimated. A notable advantage of this methodology is its capacity to facilitate rapid safety assessments while remaining independent of a broad spectrum of measurements. Various mathematical models are available to calculate the combined dermal and respiratory exposures associated with using cosmetic sprays, as well as to estimate the total systemic exposure for a given substance, essential for risk assessment.

The prevalent methodologies employed for the safety assessment of inhalation exposure predominantly rely on animal testing, with guinea pigs, mice, and rats serving as the primary animal models for screening inhalation exposure. Risk assessment programs rely on a margin of safety or margin of exposure calculation, which juxtaposes a human whole-body exposure dose against a no-observed-effect level (NOEL) or a no-observed-adverse-effect level (NOAEL) derived from an appropriate animal model. The guinea pig is favored for toxicological evaluations of inhalation exposures, as it facilitates observing pulmonary responses during the assessment process [14]. Initially, rats and mice were employed for allergen identification in cosmetic skin testing; however, many respiratory sensitizers also yield positive results in these tests. This presents a challenge, as it becomes difficult to differentiate between skin and respiratory sensitizers in practical applications [7,22]. Furthermore, respiratory sensitization can be evaluated in experimental animals, such as rats or guinea pigs, by monitoring various respiratory parameters, including the respiratory rate, inspiratory and expiratory flow rates, and expiratory intervals, either through inhalation or following provocation in the respiratory tract [23,24].

As animal testing has been prohibited in the European Union and numerous other countries in recent years, and in light of the introduction of the “3Rs” principle to protect animal rights, the pursuit of effective alternative testing methods continues. Given the similarities between skin sensitization and respiratory sensitization, many in vitro tests originally designed for skin sensitization have been adapted to assess respiratory sensitization resulting from inhalation exposures. For instance, many respiratory sensitizers exhibit reactivity in direct peptide tests, but in vitro methodologies often struggle to differentiate between sensitizers and non-sensitizers [25,26]. Specifically, while most respiratory allergens demonstrate reactivity in direct peptide response tests, it remains challenging to ascertain whether the allergen affects the skin or the respiratory tract within the context of in vitro testing, indicating a need for further investigation. Additionally, computer modeling has emerged as a valuable tool for screening inhalation exposure assessments, with several models proposed for estimating such exposures [27,28,29,30]. Nevertheless, the practical application of these models is limited, as approximately two-thirds of chemicals cannot be predicted with accuracy [31]. Rapid tests are increasingly being employed in the analysis of cosmetic toxicity. For instance, immunoassay techniques facilitate the quick identification of preservatives such as parabens—widespread additives in cosmetic formulations—allowing for the prompt assessment of associated toxicity risks. Despite their widespread application for evaluating the toxicity of cosmetic raw materials, rapid tests predominantly focus on soluble compounds. They exhibit limitations in assessing the potential risks posed by inhalable particles, particularly regarding their role in respiratory sensitization and the toxicity of insoluble particulates.

Tools such as organoids demonstrate significant potential for risk assessment, as they more accurately simulate the human body, thereby enhancing the evaluation of safety risks associated with inhalation exposure.

## 4. Safety Assessment of Lung Organoids and Their Application in Inhalable Exposed Cosmetics

### 4.1. Organoids and Their Applications

Organoids are three-dimensional structures derived from stem cells, precursor cells, and/or differentiated cells. These structures can self-organize through interactions between cells and the extracellular matrix, thereby recapitulating the native tissue structure and function in vitro [32]. The construction of organoids is contingent upon the presence of an active stem-cell population and the subsequent expansion of cell cultures over time, as well as the influence of the culture environment on the three-dimensional architecture and sustained growth of the organoids [33,34]. The most widely utilized method for developing organoids involves seeding cells in non-adhesive well plates designed with U-shaped or V-shaped bottoms. This approach capitalizes on the synergistic effects of gravity and centrifugation to promote cell aggregation, ultimately facilitating the cultivation of organoids [35].

Currently, organoids are employed in various fields, including precision medicine and drug discovery, elucidating genetic mechanisms, assessing drug efficacy and side effects, and repairing and reconstructing organs. Organoids serve as powerful disease models in mechanistic studies across a spectrum of pathologies, including genetic, infectious, and metabolic diseases, as well as various cancers. Their utility is particularly pronounced for diseases that are challenging to simulate effectively using traditional animal models. For instance, Huang et al. [36] demonstrated that retinal organoids can faithfully recapitulate the key features of X-linked juvenile retinoschisis, providing insights into the underlying mechanisms of this condition. Similarly, Taylor et al. [37] developed adipose organoids, incorporating immune cells to investigate how pro-inflammatory microenvironments stimulate lipolysis in adipocytes under insulin-resistant conditions. Moreover, numerous studies have utilized organoids to tackle critical issues such as tumor heterogeneity, immune evasion, drug resistance, and drug sensitivity [38,39,40], yielding promising results. Organoid models have also shown promise in generating cell types suitable for transplantation, including those derived from the liver, pancreas, retina, and kidney. Notably, intestinal organoids cultured from healthy intestinal mucosal stem cells from ulcerative colitis patients have successfully undergone autologous transplantation [41], demonstrating their potential for regenerative medicine applications. In addition, organoids have been employed to assess the health risks associated with various toxins. Research has indicated the cardiotoxic effects of polystyrene microplastics in cardiac organoids [42], the hepatotoxic impacts of polystyrene microparticles in hepatic organoids [43], and the nephrotoxic effects of hydroxylated fifth-generation PAMAM dendrimer nanoparticles in renal organoids [44]. Organoids derived from the lung, intestine, and other tissues have emerged as valuable models for evaluating the toxicity of per- and polyfluoroalkyl substances (PFAS) [45]. These findings underscore the relevance of organoids in toxicological studies.

The application of organoids has also been expanded to the research, development, and safety evaluation in the cosmetics field. Recent studies [46] have demonstrated that skin organoids, engineered with high precision through the use of microchannels and micropores, successfully replicate the intricate architecture of human skin, including the distinct epidermal and dermal layers. This advancement holds significant promise for cosmetic applications, particularly in assessing skin sensitization. Due to their epidermal barrier that closely resembles that of human skin, skin organoids are capable of demonstrating the absorption of skin lightening agents, evaluating skin irritation, investigating melanin inhibition, and analyzing various interactions among skin cells, thereby providing comprehensive assessment data [47]. Furthermore, the viability of tissue, inflammatory markers, and other indicators within skin organoids can effectively illustrate the damage induced by ultraviolet (UV) irradiation and evaluate the efficacy of corresponding sun protection measures [48]. There is also one study linking skin and liver organoid chips to investigate the toxicity and metabolic characteristics of the hair dye 4-amino-2-hydroxytoluene (AHT) [49], reproduce its toxic metabolic profiles in the human body, and evaluate its safety.

In summary, organoids not only enhance our understanding of complex disease mechanisms but also represent a promising platform for risk assessment and cell-based therapies, highlighting their versatile role in both basic and applied research. Future explorations should focus on refining organoid technologies to broaden their applications and efficacy in more aspects.

### 4.2. Preparation of Lung Organoids and Model Evaluation

**Figure 2:**
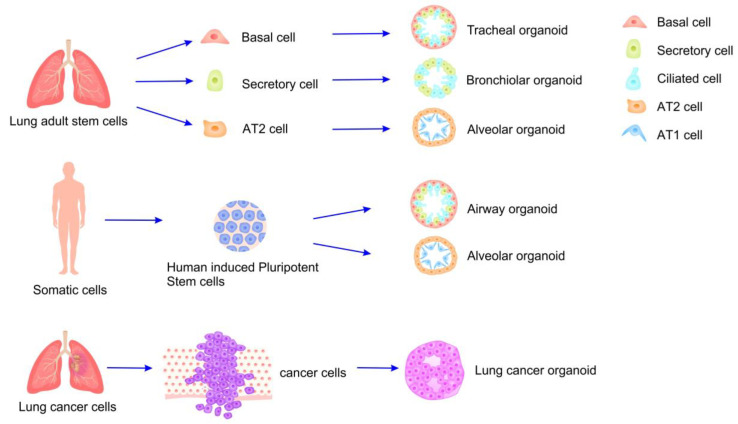
Cell types of human lung organoids.

Lung organoids are cultivated from various cell types (Figure 2), including human stem cells and alveolar epithelial progenitor cells [50,51]. These stem cells are appropriately differentiated to produce a range of lung-specific cell types, such as myofibroblasts, basal cells, functional alveolar epithelial cells (including alveolar type 1 (AT1) and type 2 (AT2) cells), and pulmonary microvascular endothelial cells [52,53]. Clinically, lung organoids are primarily utilized for investigating lung diseases [54,55]. Among these, lung cancer organoid models serve as a prominent framework for assessing the efficacy of various chemotherapeutic agents on distinct cancer mutations and for conducting drug toxicity analyses by cultivating organoids representative of different lung cancer subtypes (e.g., small-cell lung cancer and non-small-cell lung cancer) [56,57]. Additionally, lung organoids are valuable in tuberculosis research, facilitating the examination of host–microbe interactions and the evaluation of drug efficacy and side effects, thereby presenting a significant alternative to animal models [58,59].

Lung organoids can be classified into two categories: scaffolded and scaffold-free culture methods [60]. The scaffolded approach employs a carrier-based system utilizing matrigel, wherein the extracellular matrix supplies essential nutrients and associated channel proteins, thereby facilitating organoid formation. Conversely, the scaffold-free method employs a medium/air interface culture that inhibits cell adhesion to the medium, thereby enhancing cell-to-cell interactions and promoting the formation of lung spheroids, which subsequently develop into organoids.

Mature lung epithelial stem cells can be cultured directly in vitro and induced to form organoids using matrigel and differentiation media. According to [61], human bronchial epithelial cells were induced using a 5% concentration of matrigel and differentiation medium. The epithelial cells demonstrated the ability to form lumens within 7 to 10 days, develop bronchial bulbs in 8 days and produce cilia within 10 to 11 days. Furthermore, the bronchial bulbs exhibited mucous cilia differentiation after 14 days. Mature lung epithelial stem cells possess robust proliferation and differentiation capabilities in vitro, and lung organoids derived from these cells can effectively replicate the structure and function of airway secretory cells or ciliated cells under in vitro conditions. This characteristic represents a significant advantage in investigating airway-related diseases. However, categorizing organoids derived from airway epithelial cells remains challenging, necessitating identifying additional cell-type markers or enhancing culture conditions to better distinguish the sources of airway epithelial cells.

**Figure 3:**
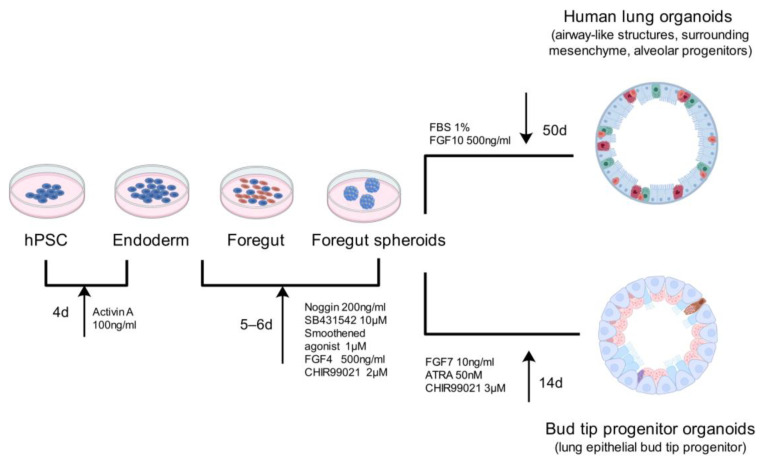
Different differentiation pathways of organoids induced by hPSCs.

Constructing lung organoids from human pluripotent stem cells (hPSCs) involves intricate signaling pathways and represents a multifaceted process (Figure 3). Initially, recombinant human activin A is used to induce hPSCs to differentiate into endoderm. Subsequently, small-molecule inhibitors facilitate the differentiation of these cells into foregut spheroids [62]. Specific growth factors and regulatory signaling pathways are then used to guide the differentiation of hPSCs into functional organoids, tailored to meet experimental requirements. For instance, applying FGF7, Chir99021, and ATRT promotes the differentiation of pre-gut spheroids into bud tip organoids, while introducing 1% FBS and a high concentration of FGF10 induces the differentiation of foregut spheroids into proximal lung organ structures [63]. hPSCs are relatively accessible, and the organoids derived from them can be extensively utilized in various pathological and physiological research studies. Furthermore, hPSCs can be modified through gene-editing techniques, such as CRISPR/Cas9, to achieve targeted modifications. However, it is important to note that hPSC-derived organoids require a complex targeted differentiation protocol established in the early stages and may exhibit epigenetic abnormalities.

Lung cancer organoids are commonly used as models for disease research and can be generated from tumor cells isolated from patient tissues. SACHS et al. [54] collected bronchoalveolar resection tissue or lavage fluid from patients diagnosed with non-small-cell lung cancer during surgical procedures. They isolated individual cells through mechanical and enzymatic methods, subsequently embedding them in type 2 matrigel extracts. These cells were then induced in a targeted manner using differentiation reagents, resulting in the successful generation of three-dimensional organoids within a few days, achieving a success rate of 94%. Lung cancer organoids are capable of retaining multiple phenotypic characteristics of tumor cells, offering significant advantages over other in vitro culture models. They play a crucial role in understanding tumorigenesis, facilitating drug screening, and predicting patient-specific drug responses. However, it is important to note that the current lung cancer organoid model lacks a comprehensive tumor microenvironment, including stromal and immune cells, which needs to be improved in future research.

### 4.3. Safety Assessment of Lung Organoids Applied in Cosmetics After Inhalation Exposure

To fulfill the requirements of in vitro studies concerning inhalation exposure, it is essential that in vitro culture systems closely replicate lung function. This necessity arises from the strong correlation between lung deposits, such as aerosols and particulate matter, and various lung cell types. Optimal in vitro models should be capable of recapitulating the partial lung defense mechanisms and the anticipated responses to exposure. Huh et al. [64] developed an in vitro system that co-cultured endothelial cells to simulate respiration. They found that the application of mechanical stress significantly influenced the cellular response to 12 nm silica particles, which were used to represent air pollutants. The findings from this study were consistent with observations from an isolated mouse model of ventilation and perfusion. Similarly, Winkler et al. [65] created tracheal organoids to investigate the effects of microplastic fibers and their deposition in inhaled air. Their research demonstrated that the organoids had adequate time to adapt to the microplastic environment, in contrast to traditional two-dimensional models. Furthermore, integrating morphological approaches with molecular techniques facilitated a comprehensive assessment of outcomes at both the organ and cellular levels, as well as a more nuanced analysis of three specific toxicological pathways: inflammation, oxidative stress, and obesity responses. The results of these studies indicate that organoids are appropriate for evaluating the potential risks associated with inhalation exposure to pollutants, particularly in the context of adverse pulmonary effects. Consequently, lung organoids may serve as valuable tools for assessing the safety of inhalation exposures to cosmetic products.

Studies have investigated the application of three-dimensional (3D) cell culture models and lung organoids for assessing hazards associated with respirable particles, akin to the safety evaluations conducted for cosmetic inhalation exposures. Huang et al. [66] demonstrated that an in vitro recombinant 3D respiratory epithelial cell model could effectively distinguish between respiratory sensitizers, skin sensitizers, and respiratory irritants. Chary et al. [67] developed an in vitro model that accurately replicated the alveolar–capillary barrier. This 3D in vitro model, cultivated at the air–liquid interface, comprised alveolar type II epithelial cells, endothelial cells, macrophage-like cells, and dendritic cells, and it proved effective in differentiating between chemical respiratory sensitizers and respiratory irritants. Mizoguchi et al. [68] introduced an innovative 3D co-culture system for the human upper respiratory epithelium, which included human respiratory epithelial cells, immature dendritic cells (DCs) and human lung fibroblasts. These components were cultured separately before being assembled into a 3D multicellular tissue model, thereby more accurately simulating in vivo conditions. This system successfully differentiated chemical respiratory sensitizers from skin sensitizers by quantifying OX40L, a critical molecule involved in Th2 differentiation in DCs. These studies indicate that 3D cell culture models possess significant potential for application in the safety assessment of inhalation exposures, as well as that identifying respiratory sensitizers in cosmetic products can be effectively achieved using these methodologies.

In addition to their role in identifying respiratory sensitizers, 3D cell culture models and lung organoids may prove beneficial in toxicologically evaluating inhaled particles and assessing lung damage resulting from respirable insoluble particles. Studies have been conducted on this topic [69,70,71]. The toxicological characterization of lung and respiratory epithelial damage induced by cigarette smoke and tobacco aerosols using lung organoids has demonstrated that these organoids are effective in evaluating cytotoxicity and other relevant characteristics. Furthermore, they replicate the inhalation exposure experienced by the human body and more accurately reflect the authentic human microenvironment. Lung organoids have also been employed in studies concerning air pollutants [72,73] and nanomaterials [51]. Applications that negatively impact lung tissue or lung cells, including effects on cell proliferation, oxidative stress, DNA damage and pro-inflammatory and pro-fibrotic responses, mimic the in vivo environment, thereby facilitating the comparison of in vivo and in vitro findings.

Applying lung organoids to evaluate inhalation exposure to cosmetic products effectively simulates the authentic microenvironment of the human body. This approach facilitates identifying respiratory allergens present in cosmetic ingredients, differentiating them from skin allergens and non-sensitizing respiratory irritants. Furthermore, it enables comprehensive toxicological assessments of both cosmetic products and their constituents. Lung organoids hold significant potential for application in the safety evaluation of inhalation exposure to cosmetics.

## 5. Future Perspectives and Conclusions

Despite the considerable promise demonstrated by the organoid field across various applications, several significant challenges and limitations persist. First, while organoid technology effectively bridges the gap between traditional cell lines and more complex in vivo models, most organoids are devoid of essential surrounding components, which are crucial for creating more physiologically relevant models that accurately reflect the complexities of human tissues. Second, the organoid industry grapples with a lack of standardization, which hampers reproducibility and the practicality of experimental results.

Current research efforts are gradually addressing these issues. For instance, studies have successfully cultivated airway organoids that can be transplanted in vivo, demonstrating the ability to proliferate and maintain function for over two years [74]. Efforts to enhance standardization have included the use of artificial intelligence (AI) for the systematic evaluation of organoids [75,76], which aims to improve their accuracy and reliability. Additionally, novel alternative materials, such as graphene oxide, have been employed in organoid culture to mitigate batch-to-batch variability [41].

The range and quantity of aerosolized cosmetic products are increasing, alongside the number of users. This trend, coupled with the potential health risks associated with these products, has garnered the attention of regulatory authorities. In instances where inhalation exposure is possible, it is imperative to evaluate the health hazards linked to such exposure and to conduct appropriate safety assessments. Organoids represent a promising approach for these assessments. Although constructing a comprehensive in vitro model of the lungs remains challenging, breakthrough developments are anticipated through the interdisciplinary integration of various technological advancements in the near future. On one side, innovations such as 3D bioprinting and microfluidics are paving the way for the construction of complex lung organoid models that incorporate multiple cell types, including airway epithelium, immune cells, and vascular endothelial cells. These models aim to more accurately replicate the human lung microenvironment, thereby providing enhanced physiological relevance. On the other side, the fusion of artificial intelligence (AI) and big data analytics promises to facilitate a high-throughput and precise evaluation of critical parameters, such as cytotoxicity and inflammatory responses, particularly in relation to inhalation exposure to cosmetic ingredients. This integration has the potential to significantly accelerate risk prediction and safety assessment processes, making these evaluations more efficient and reliable. Additionally, the ongoing development of standardized culture systems and innovative biomaterials is expected to markedly improve the stability and reproducibility of lung organoid models. By addressing these challenges, lung organoids are poised to gradually supplant traditional animal experimentation, emerging as core tools for the safety assessment of inhalation exposure to cosmetic products.

## Figures and Tables

**Table 1:** Particle diameters and their deposition sites.

Particle Diameter (μm)	Sedimentation Site
>100	hard to enter the respiratory tract
10~100	upper respiratory tract
5~10	bronchiole, pulmonary acinus
<5	pulmonary acinus

**Table 2:** Common ingredients in cosmetic sprays.

Ingredient	Examples
Solvent	Water, oil, ethanol, isopropanol, etc.
Propellant	Propane, butane, etc.
Humectant	Glycerin, hyaluronic acid, propylene glycol, etc.
Active ingredients	Vitamins, plant extracts, peptides, etc.
Preservative	Phenoxyethanol, parabens, etc.
Fragrance and pigment	Natural fragrance, synthetic fragrance, natural pigment, synthetic pigment, etc.
Other additives	Thickener, antioxidant, etc.

**Table 3:** Conservative default data for aerosol exposures in different regions.

Cosmetic Type	Daily Exposure (g/Day)		
	US	Europe	Netherlands
Spray Perfume	0.53		0.1
Deodorant/Antiperspirant (spray)		0.69	0.4
Hair-Styling Spray	5.18		0.4
References	[19]	[20]	[21]
